# Fatal Myocarditis Following Adjuvant Immunotherapy: A Case Report and Literature Review

**DOI:** 10.3390/ijms262311646

**Published:** 2025-12-01

**Authors:** Nanteznta Torounidou, Melina Yerolatsite, Vasileios Bouratzis, Anna-Lea Amylidi, George Zarkavelis, Katerina K. Naka, Paraskevi V. Voulgari, Stergios Boussios

**Affiliations:** 1 Department of Medical Oncology, University Hospital of Ioannina, 45500 Ioannina, Greece; m.yerolatsite@gmail.com (M.Y.);; 2 Second Department of Cardiology, University Hospital of Ioannina, Stavros Niarchos Avenue, 45500 Ioannina, Greece; v.bouratzis@gmail.com (V.B.); anaka@uoi.gr (K.K.N.); 3 Society for Study of Clonal Heterogeneity of Neoplasia (EMEKEN), 45445 Ioannina, Greece; 4 Faculty of Medicine, School of Health Sciences, University of Ioannina, 45110 Ioannina, Greece; 5 Department of Rheumatology, School of Health Sciences, Faculty of Medicine, University of Ioannina, 45110 Ioannina, Greece; pvoulgar@uoi.gr; 6 Faculty of Medicine, Health and Social Care, Canterbury Christ Church University, Canterbury CT1 1QU, UK; 7 Faculty of Life Sciences & Medicine, School of Cancer & Pharmaceutical Sciences, King’s College London, Strand, London WC2R 2LS, UK; 8 Department of Research and Innovation, Medway NHS Foundation Trust, Gillingham Kent ME7 5NY, UK; 9 AELIA Organization, 9th Km Thessaloniki—Thermi, 57001 Thessaloniki, Greece

**Keywords:** myocarditis, cardiotoxicity, immune checkpoint inhibitors (ICIs), immune-related adverse events (irAEs), fatal side effects

## Abstract

Immune checkpoint inhibitors (ICIs) have revolutionized cancer therapy by enhancing the immune response against tumors. However, they can cause immune-related adverse events (irAEs), including rare but potentially fatal myocarditis. We describe a 71-year-old man with stage IIIA lung adenocarcinoma treated with adjuvant pembrolizumab who developed severe ICI-associated myocarditis. Despite early diagnosis, treatment with intensive immunosuppression and mechanical support, he suffered fatal cardiac complications. A systematic review of the literature up to May 2025 identified 44 cases of ICI-associated myocarditis. Data on clinical features, diagnostics, treatment, and outcomes were extracted and analyzed. Most cases involved older patients with lung cancer treated with pembrolizumab or nivolumab. Onset varied from days to years after therapy initiation. Presentations included dyspnea, chest pain, arrhythmias, and elevated cardiac biomarkers. The biopsy showed T-cell and macrophage infiltration. High-dose corticosteroids were the primary treatment; additional immunosuppressants were used in cases that were refractory. Mortality was 45%, mainly due to cardiac failure and sepsis. Discussion: ICI-associated myocarditis arises from immune dysregulation affecting cardiac tissue, potentially involving shared antigens and systemic inflammation. Early detection and aggressive immunosuppression are crucial but often insufficient, resulting in high mortality. This underscores the urgent need for a better understanding of pathogenesis and the development of effective management strategies to improve patient outcomes. Finally, a multidisciplinary approach is important to improve outcomes in ICI-associated myocarditis.

## 1. Introduction

Immune checkpoint inhibitors (ICIs) have marked a major breakthrough in oncology by boosting the immune system’s ability to attack tumors through the inhibition of regulatory pathways such as cytotoxic T-lymphocyte-associated protein 4 (CTLA-4) and programmed death-1 (PD-1)/programmed death-ligand 1 (PD-L1). There are different types of ICIs, including CTLA-4 inhibitors (ipilimumab), PD-1 inhibitors (nivolumab, pembrolizumab, cemiplimab), and PD-L1 inhibitors (atezolizumab, avelumab, durvalumab). Despite their clinical success, ICIs can trigger immune-related adverse events (irAEs), affecting organs such as the gastrointestinal tract, skin, liver, and endocrine glands, particularly when used in combination therapies. Although most irAEs respond to treatment interruption and immunosuppression, cardiac side effects such as myocarditis can be severe and potentially fatal [1,2,3].

Myocarditis is the most common and serious form of ICI-associated cardiotoxicity, though other cardiac complications such as cardiomyopathy, arrhythmias, acute coronary syndrome, and vasculitis have also been reported [4,5,6]. The incidence of immune myocarditis among cancer patients enrolled in clinical trials receiving ICIs has been reported to range from 0.06% to 1% [7]. Moreover, a study by Johnson et al. demonstrated a significant difference in incidence between treatment regimens: 0.06% in patients treated with nivolumab alone and 0.27% in those receiving combination therapy with nivolumab and ipilimumab [8]. The exact mechanism remains unclear, but shared antigens between tumor and heart tissues may play a role. Additionally, animal studies suggest that CTLA-4, PD-1, and PD-L1 have cardioprotective effects, and their inhibition may increase the heart’s vulnerability to immune damage [5,6,7].

Recent guidelines from the European Society of Cardiology (ESC) and the European Society for Medical Oncology (ESMO) offer clear recommendations for diagnosing and managing ICI-associated myocarditis [9,10]. Similarly, the American Society of Clinical Oncology (ASCO) has published guidelines for managing irAEs in patients treated with ICIs [11]. The 2022 ESC Cardio-Oncology Guidelines stress early detection and prompt treatment, advising immediate cessation of ICI therapy upon suspicion of myocarditis and initiation of high-dose corticosteroids (e.g., methylprednisolone 500–1000 mg IV daily) for 3–5 days or until improvement. If needed, additional immunosuppressants such as infliximab, antithymocyte globulin (ATG), or intravenous immunoglobulin may be used. The guidelines also emphasize the importance of multidisciplinary care tailored to the patient. Similarly, the ESMO Clinical Practice Guideline recommends stopping ICI therapy and initiating high-dose corticosteroids promptly, along with close monitoring and supportive care, with consideration of additional immunosuppressive treatments when appropriate [9,10].

Here, we present a case report from our hospital and the fatal outcome of this patient despite the timely and coordinated efforts of oncologists, cardiologists, and rheumatologists. Additionally, we conducted a literature review to identify all relevant articles reporting similar cases.

## 2. Detailed Case Description

A 71-year-old man presented to the emergency department with palpitations, generalized weakness, fatigue, and easy exhaustion with minimal exertion for the past three days. He denied chest pain or shortness of breath. His medical history included arterial hypertension, dyslipidemia, and a 70-pack-year smoking history. In 2003, he underwent a radical prostatectomy as definitive treatment for prostate cancer.

**Figure 1:**
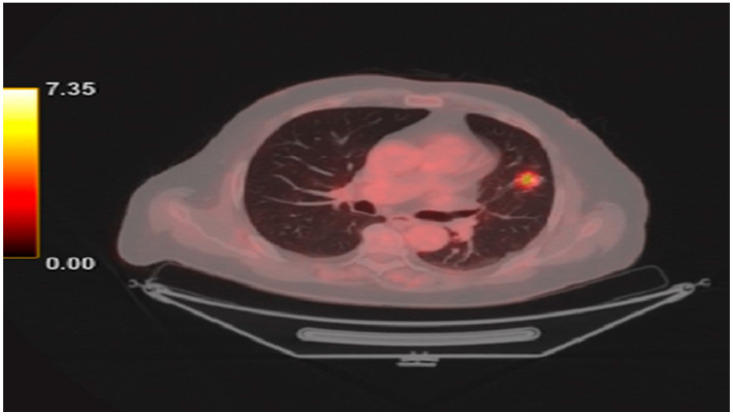
PET/CT demonstrating a hypermetabolic lesion in the glottic region, corresponding to the primary site of disease.

In May 2024, he underwent a left upper lobectomy for stage IIIA (pT2N2) lung adenocarcinoma characterized by a Ki-67 index of 20–25%, PD-L1 expression of 10%, and mutations in Kirsten rat sarcoma viral oncogene homolog (KRAS) (exon 2), serine/threonine kinase 11 (STK11), and tumor protein 53 (TP53). He subsequently received adjuvant chemotherapy with cisplatin and pemetrexed. Sixteen days prior to presentation, he received his first cycle of adjuvant pembrolizumab according to the PEARLS/KEYNOTE-091 trial [12]. Figure 1 shows the positron emission tomography/computed tomography (PET/CT) scan performed for diagnosis.

The patient’s blood pressure was 140/78 mmHg, and his heart rate was 104 beats per minute with a regular rhythm. His body temperature was 36.4 °C. On physical examination, there was no lower limb swelling or jugular vein distension. S1 and S2 were audible, and no murmurs were heard on cardiac auscultation. Lung examination revealed bilateral wheezing.

**Figure 2:**
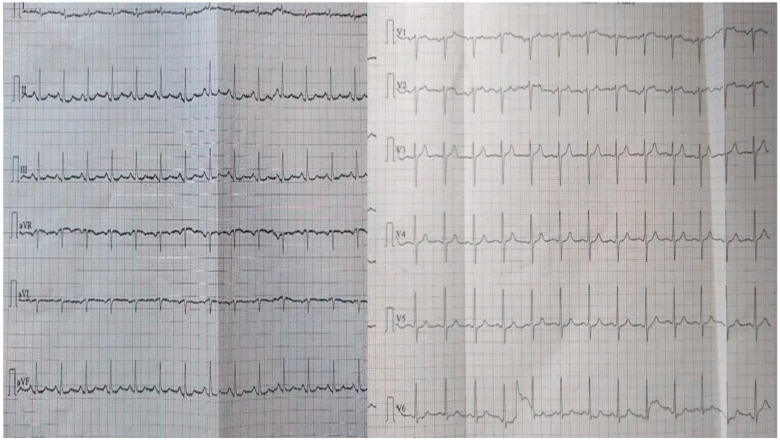
Pre-admission electrocardiogram (ECG) obtained prior to arrival in the emergency department, documenting the patient’s baseline cardiac rhythm.

**Figure 3:**
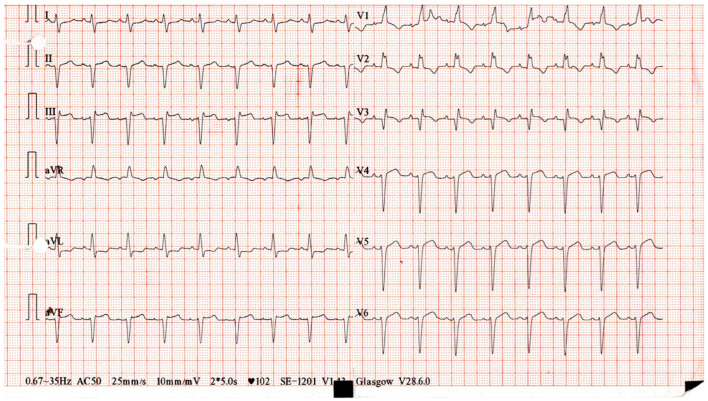
ECG in ER showing sinus tachycardia with a newly diagnosed RBBB.

The electrocardiogram (ECG) showed sinus tachycardia with a newly diagnosed right bundle branch block. Figure 2 illustrates the ECG recorded before the patient arrived at the emergency unit (EU), and Figure 3 shows the ECG obtained upon arrival. Echocardiography revealed a right ventricle of normal size but with impaired function.

On evaluation in the EU, laboratory tests revealed a hemoglobin level of 16.1 g/dL (normal for men: 13.5–17.5 g/dL), a white blood cell count of 12,550/μL (normal: 4000–11,000/μL), and a platelet count of 234,000/μL (normal: 150,000–450,000/μL). Renal function was within normal limits, with a creatinine level of 0.73 mg/dL (normal: 0.6–1.2 mg/dL) and urea at 34 mg/dL (normal: 11–54 mg/dL). Liver enzymes were markedly elevated, with aspartate aminotransferase (AST) at 566 IU/L (normal: 10–35 IU/L) and alanine aminotransferase (ALT) at 277 IU/L (normal: 10–35 IU/L). Electrolytes were within normal ranges, with sodium at 137 mEq/L (normal: 136–146 mEq/L) and potassium at 4.84 mEq/L (normal: 3.5–5.3 mEq/L). Lactate dehydrogenase (LDH) was significantly elevated at 979 U/L (normal: 115–230 U/L). Coagulation parameters showed an international normalized ratio (INR) of 1.15 (normal: 0.8–1.2). High-sensitivity cardiac troponin I was markedly elevated at 13,983.1 pg/mL (normal: <14 pg/mL), along with creatine kinase (CK) at 7166 IU/L (normal for men: 40–190 IU/L) and CK-MB at 173 U/L (normal: <25 U/L). D-dimer levels were elevated at 2.88 μg/mL (normal: <0.5 μg/mL), and C-reactive protein (CRP) was significantly elevated at 79 mg/L (normal: <6 mg/L). Arterial blood gas analysis revealed a lactate level of 0.9 mmol/L (normal: 0.5–2.2 mmol/L).

**Figure 4:**
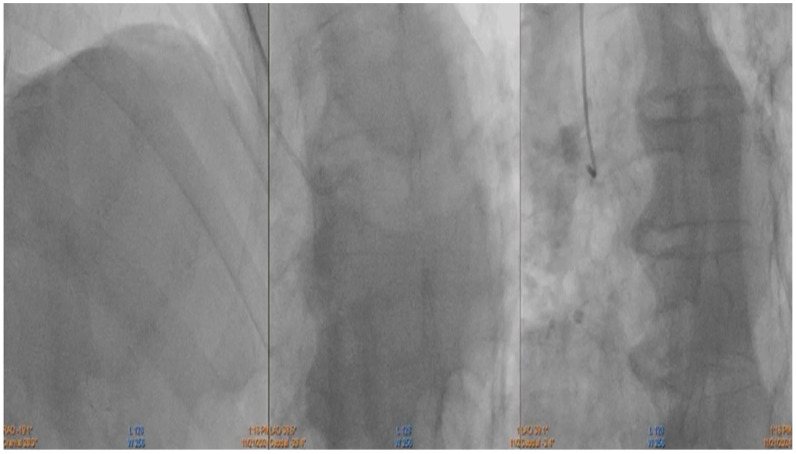
Coronary angiography showing no significant stenosis in the coronary arteries.

The patient was admitted to the coronary care unit due to a high suspicion of non–ST-elevation myocardial infarction (NSTEMI). Initially, acute coronary syndrome was ruled out by performing an emergent coronary angiography, which revealed no significant stenosis in the coronary arteries (Figure 4).

**Figure 5:**
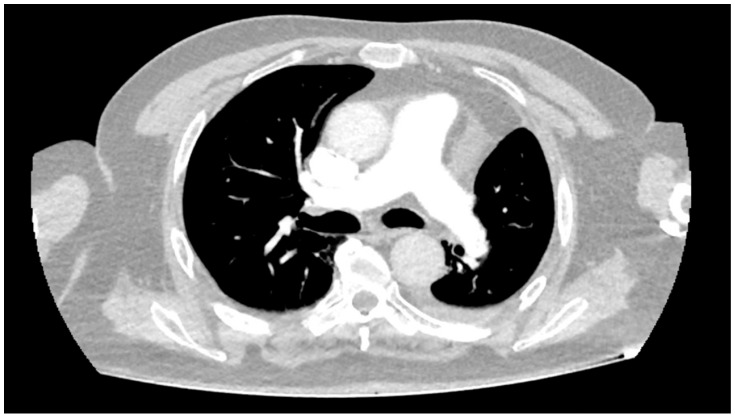
CTPA negative for pulmonary embolism.

Subsequently, a computed tomography pulmonary angiography (CTPA) was performed, which also ruled out pulmonary embolism (Figure 5).

A full viral blood workup, including a coronavirus disease 2019 (COVID-19) test, as well as tests for tuberculosis and blood cultures, was obtained, all of which eventually returned negative. Cardiac magnetic resonance imaging (MRI) performed the next day confirmed the diagnosis. Specifically, the MRI showed diffuse delayed enhancement of the myocardium throughout all walls of the left ventricle, with particularly patchy, almost transmural enhancement observed in the apical inferior and apical regions. These findings were suggestive of myocarditis related to immune checkpoint inhibitor therapy, according to the modified Lake Louise criteria [10].

Since an endomyocardial biopsy could not be performed at our center, the diagnosis of ICIs myocarditis was made according to the 2022 ESC Cardio-Oncology Guidelines. Diagnosis requires elevated troponin (after ruling out other causes such as acute infectious myocarditis and acute coronary syndrome) plus either one major criterion (diagnostic cardiac MRI) or at least two minor criteria. In this case, the patient presented with a clinical syndrome, immune-related adverse events, and suggestive cardiac MRI findings (three minor criteria), accompanied by elevated troponin levels. Given the recent initiation of immunotherapy with pembrolizumab, the patient was diagnosed with immunotherapy-induced myocarditis and was immediately started on high-dose corticosteroid therapy (1 g IV methylprednisolone for 3–5 consecutive days), along with optimal cardiovascular medical therapy, including ramipril 5 mg once daily and bisoprolol 5 mg once daily.

**Figure 6:**
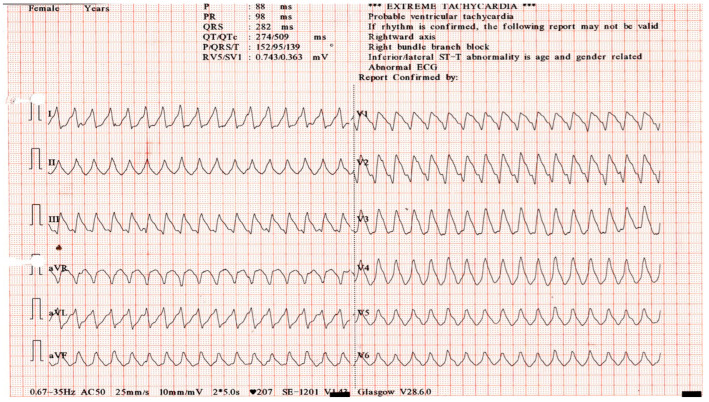
ECG recorded during hospitalization showing the occurrence of ventricular tachycardia (VT), a serious complication.

**Figure 7:**
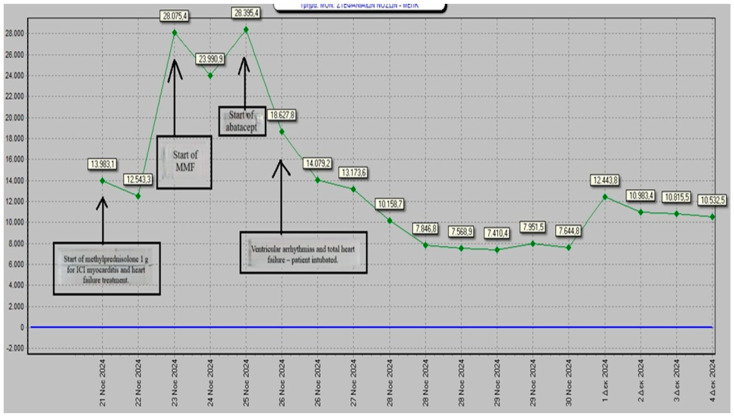
Graph illustrating changes in troponin levels throughout the hospital stay.

On day 3, because troponin levels had not decreased by 50%, complicated myocarditis was diagnosed, and a rheumatology consultation was requested. Following the rheumatologist’s advice, mycophenolate mofetil was started at 500 mg twice daily for 3 days and then increased to 1000 mg twice daily. This escalation was consistent with ESC and ESMO recommendations, which advise introducing second-line immunosuppression when high-dose corticosteroids fail to achieve clinical or biochemical improvement and the patient shows signs of clinical worsening. On day 6, the patient developed conduction abnormalities with alternating blocks, ventricular arrhythmias, and biventricular dysfunction, with a reduced left ventricular ejection fraction (LVEF) of 35%. Troponin levels continued to rise, and blood gas analysis showed elevated lactate levels. Figure 6 shows the ECG recorded during hospitalization, demonstrating the occurrence of a serious complication—ventricular tachycardia (VT)—while Figure 7 illustrates the changes in troponin levels throughout the hospital stay.

Antiarrhythmic therapy with amiodarone and lidocaine was initiated, but due to hemodynamic instability and hypoxemia, the patient was intubated and supported hemodynamically with an intra-aortic balloon pump and vasopressors. Additionally, because of continued clinical and biomarker worsening, along with the development of malignant arrhythmias and hemodynamic instability, abatacept was administered intravenously at a dose of 750 mg, with plans for subsequent doses to be given subcutaneously. Despite third-line immunosuppressive therapy with abatacept, the patient did not respond to treatment. He subsequently developed renal failure, and continuous hemofiltration was initiated. Unfortunately, he passed away due to sepsis followed by multi-organ failure after 15 days of hospitalization. Table 1 provides a summary of the timeline of the patient’s clinical events.

## 3. Review of the Literature

### 3.1. Methods

#### 3.1.1. Search Strategy

A structured literature search was conducted to identify all published case reports and case series describing myocarditis associated with ICI therapy. Searches were performed in PubMed, Scopus, and the Cochrane Library from database inception to May 2025. To maximize the sensitivity of the search, a broad and inclusive algorithm was used: (neoplasia OR cancer OR carcinoma) AND (immunotherapy OR immune checkpoint OR ICIs) AND (myocarditis OR myocardial involvement OR myotoxicity OR cardiac immune-related adverse events OR cardiological immune-related side effects). No filters related to study design, publication year, or article type were applied to avoid missing relevant reports. To further ensure completeness, the reference lists of all retrieved articles were manually screened for additional eligible publications. Two of the three databases returned relevant results, whereas the third did not generate any articles meeting the criteria.

#### 3.1.2. Study Inclusion and Exclusion Criteria

Studies were included if they met the following criteria: (1) described myocarditis attributed to ICI therapy; (2) presented original patient data in the form of a case report or case series; (3) involved adult patients receiving ICIs; (4) were published in English; (5) provided full-text availability. We excluded non-English publications, non-human studies, mechanistic reports without patient cases, and articles describing myocarditis caused by infectious, ischemic, toxic, or autoimmune etiologies unrelated to ICIs. Reports focusing solely on other cardiac immune-related adverse events without myocarditis were also excluded.

#### 3.1.3. Article Selection and Data Extraction

**Figure 8:**
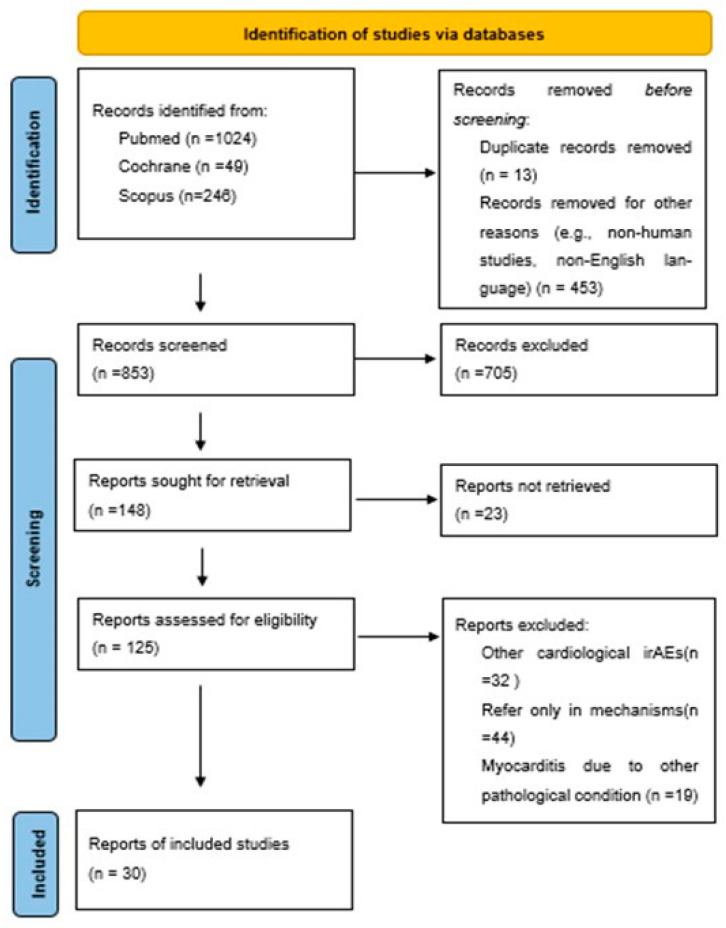
Flowchart depicting the search strategy followed in our analysis.

The initial database search yielded a total of 1319 records (PubMed n = 1024; Cochrane n = 49; Scopus n = 246). After removing 13 duplicate records and 453 additional articles for reasons such as non-English language, non-human populations, or unrelated subject matter, 853 articles remained for title and abstract review. Of these, 705 were excluded because they did not meet predefined eligibility criteria. An additional 23 studies could not be retrieved. The remaining 125 full-text articles were reviewed in detail, and 30 publications reporting 45 individual cases of ICI-associated myocarditis were identified as eligible and included in the final analysis. The selection process is summarized in a PRISMA-style flow diagram (Figure 8).

Data extraction was performed independently by two reviewers (N.T and M.Y.) using a predefined structured form. Extracted variables included demographics, cancer type, ICIs regimen, timing of myocarditis onset, symptoms, ECG findings, cardiac biomarkers, imaging results, biopsy findings, treatment strategies, cardiac complications, and clinical outcomes. Disagreements were resolved through discussion and consensus.

Quality assessment was conducted for all included case reports and case series to ensure reliability of the extracted data. Each article was evaluated for clarity of diagnosis, completeness of clinical information, adherence to recognized definitions of ICI-associated myocarditis, and adequacy of reported investigations and follow-up. Formal scoring tools were not applied due to the nature of case-based evidence, but emphasis was placed on diagnostic transparency and completeness of reporting. Only studies with sufficient information to support the diagnosis were included.

Due to the heterogeneity of the included reports, particularly regarding clinical presentation, diagnostic evaluation, and treatment approaches, a quantitative meta-analysis was not feasible. Therefore, a descriptive narrative synthesis was performed. Findings across cases were summarized thematically and organized in tabular form (Table 2), highlighting the most common and less common clinical features and diagnostic pattern.

## 4. Results

We identified 30 relevant articles presenting case reports of immune-associated myocarditis. Although some articles described more than one case, the total number of reported cases was 45 [12,13,14,15,16,17,18,19,20,21,22,23,24,25,26,27,28,29,30,31,32,33,34,35,36,37,38,39,40,41].

Approximately 57.8% of the patients were male (26/45). The mean age was 66.68. The youngest patient was 35 years old [12], and the oldest was 89 years old [41]. The mean time from the start of immunotherapy to the onset of myocarditis was 103 days. There were two cases in which myocarditis appeared much later—2.5 years and 8 months after the initiation of immunotherapy [24,33]. If these two outliers are excluded, the mean time to onset was 67 days. The earliest presentation occurred 3 days after the initiation of ICIs [35], and the latest was at 2.5 years. None of the patients had autoimmune diseases. Most of the patients had hypertension (22/45) and diabetes (13/45). Atrial fibrillation was present in three patients, and coronary artery disease was also reported in three patients. Seven patients had no significant medical history, and for five patients, data on medical history were not available [13,14,15,16,17,18,19,20,21,22,23,24,25,26,27,28,29,30,31,32,33,34,35,36,37,38,39,40,41,42].

The most common type of cancer was lung cancer (17/45), followed by renal cell carcinoma (8/45). In most cases, pembrolizumab was the immune checkpoint inhibitor used (12/45), followed by nivolumab (10/45) and the combination of ipilimumab and nivolumab (9/45). Immunotherapy was administered either in the adjuvant or metastatic setting [13,14,15,16,17,18,19,20,21,22,23,24,25,26,27,28,29,30,31,32,33,34,35,36,37,38,39,40,41,42].

The most common presenting symptoms of immune-associated myocarditis were dyspnea (shortness of breath), fatigue, chest pain or discomfort, myalgia, and palpitations. These symptoms often appeared alone or in combination and reflect the cardiac, respiratory, and systemic involvement typical of immune-mediated toxicity. Neurological symptoms such as ptosis, diplopia, blurred vision, and weakness were also noted in several cases, indicating a possible overlap with myositis or myasthenia-like syndromes [13,14,15,16,17,18,19,20,21,22,23,24,25,26,27,28,29,30,31,32,33,34,35,36,37,38,39,40,41,42].

In contrast, the rarest symptoms included cardiac arrest, right-sided facial droop, presyncope, asymptomatic presentation with only mild CK elevation, watery diarrhea with frequent premature ventricular contractions (PVCs), and severe asthenia due to subcutaneous tumor infiltration. These atypical manifestations highlight the broad and sometimes subtle clinical spectrum of myocarditis associated with immune checkpoint inhibitors [13,14,15,16,17,18,19,20,21,22,23,24,25,26,27,28,29,30,31,32,33,34,35,36,37,38,39,40,41,42].

At presentation in the emergency department, the most common electrocardiographic findings included ST-segment elevation in various leads (anterolateral, inferolateral, V1–V6, II, III, and aVF), sinus tachycardia, and right bundle branch block (RBBB). These findings typically indicated acute myocardial injury or inflammation. Other frequent abnormalities were atrial fibrillation, PVCs, and T-wave inversions [13,14,15,16,17,18,19,20,21,22,23,24,25,26,27,28,29,30,31,32,33,34,35,36,37,38,39,40,41,42].

Rare but severe ECG presentations included complete atrioventricular block (CAVB), ventricular tachycardia (VT), wide QRS complex arrhythmias, QT prolongation, alternating bundle branch blocks, asystole, and episodes of third-degree heart block with junctional escape rhythms. A few patients had normal ECGs or showed only minor changes such as lateral ST-segment depression or nonspecific ST-T wave abnormalities. This broad spectrum of ECG presentations highlights the variable cardiac involvement of immune-associated myocarditis at initial emergency evaluation [13,14,15,16,17,18,19,20,21,22,23,24,25,26,27,28,29,30,31,32,33,34,35,36,37,38,39,40,41,42].

At presentation, LVEF varied widely among patients with immune-associated myocarditis. The most common findings included preserved or mildly reduced LVEF (50–60%), often accompanied by mild left ventricular hypertrophy (LVH), mild-to-moderate left atrial enlargement, and mild regurgitation of the aortic, mitral, and tricuspid valves. Many patients exhibited normal wall motion or mild diastolic dysfunction with preserved systolic function. Several cases also showed right ventricular dilation with preserved right ventricular systolic function [13,14,15,16,17,18,19,20,21,22,23,24,25,26,27,28,29,30,31,32,33,34,35,36,37,38,39,40,41,42].

Conversely, severe left ventricular systolic dysfunction with markedly reduced LVEF (ranging from 10% to 35%) was noted in a subset of patients, often accompanied by diffuse hypokinesis, apical thrombi, and pericardial effusions. Some patients had new wall motion abnormalities, akinetic or hypokinetic segments, and signs of right heart failure such as right atrial dilation and hepatic vein reflux. A few cases reported hyperdynamic basal segments despite an overall reduced LVEF [13,14,15,16,17,18,19,20,21,22,23,24,25,26,27,28,29,30,31,32,33,34,35,36,37,38,39,40,41,42].

In some patients, echocardiograms were unremarkable, with normal LVEF and no regional wall motion abnormalities, while in others, data were not available. These findings highlight the heterogeneity of cardiac involvement in immune checkpoint inhibitor–associated myocarditis, ranging from subtle functional changes to severe cardiomyopathy. In half of the patients, MRI data were not available. Among the remaining cases, only three had MRI findings that were negative for myocarditis [18,19,41].

At the emergency department, patients exhibited a wide range of cardiac biomarker levels, with troponin often markedly elevated, indicating ongoing myocardial injury. CK levels varied considerably, from normal to highly increased, reflecting muscle damage, while CK-MB values, when available, were frequently elevated. BNP or NT-proBNP levels ranged from normal to significantly increased, suggesting cardiac stress or heart failure [13,14,15,16,17,18,19,20,21,22,23,24,25,26,27,28,29,30,31,32,33,34,35,36,37,38,39,40,41,42].

In addition, only 15 out of 44 cases underwent myocardial biopsy due to the difficulty of the procedure and safety concerns for the patients. In the case report by Hu Y., the biopsy revealed degeneration of myocardial cells and an inflammatory infiltrate consisting predominantly of CD3^+^CD4^+^CD8^+^ cells in the myocardium [19]. In the case report by Le Bras, the biopsy showed abnormalities suggestive of anti-PD-L1–related cardiac involvement, and the hypertrophy could be secondary to arterial hypertension [22]. In the study by Izumi, which presented four different case reports, myocardial biopsy was performed in all cases. In the first case, the biopsy revealed CD3^+^ T-cells (with CD8 predominating over CD4), CD68^+^ histiocytes, a few CD20^+^ B-cells, a few eosinophils, and high expression of Tenascin-C and PD-L1. The second case showed a similar pattern with CD3^+^ T-cells (CD8 > CD4), CD68^+^ histiocytes, a few CD20^+^ B-cells, neutrophils and eosinophils, and high levels of Tenascin-C and PD-L1. In the third case, the biopsy demonstrated CD3^+^ T-cells with equal CD4 and CD8 expression, CD68^+^ histiocytes, a few CD20^+^ B-cells, and moderate expression of Tenascin-C and PD-L1. Finally, in the fourth case, the biopsy showed CD3^+^ T-cells (CD8 > CD4), rare CD68^+^ histiocytes and CD20^+^ B-cells, with low expression of Tenascin-C and PD-L1 [23]. In the article by Lewis I., the myocardial biopsy showed distinct focal and diffuse interstitial fibrosis, suggestive of inflammation that likely preceded the out-of-hospital cardiac arrest (OHCA) subclinically by several months. In various areas, myocyte necrosis was observed in association with numerous CD3^+^ T-cells (100/mm^2^), a large number of CD68^+^ macrophages expressing MHC II (200/mm^2^), and scattered CD20^+^ B-lymphocytes, with no eosinophilic granulocytes or multinucleated giant cells present [24]. In the case report by Miyauchi Y., a myocardial biopsy performed during the acute phase showed marked lymphocytic infiltration, predominantly composed of CD4^+^ cells. A repeat biopsy on day 32 of steroid treatment revealed a mixed population of CD4^+^ and CD8^+^ T cells along with numerous histiocytes/macrophages, consistent with chronic smoldering myocarditis [30]. In the case report by Naganuma K., the biopsy revealed focal infiltration of inflammatory cells, predominantly CD8-positive T cells, around myocardial cells, without evidence of myocardial necrosis. Additionally, limited PD-L1 expression was observed in some myocardial cells [33]. In the article by Sakaguchi H., two different cases are presented. In one of them, a myocardial biopsy was performed. Histological examination revealed myocardial fibrosis and lymphocytic infiltration despite high-dose corticosteroid treatment, suggesting that corticosteroid monotherapy was insufficient for managing the myocarditis [36]. In the case report by Zadok O., the myocardial biopsy showed fulminant myocarditis with abundant lymphocytes, eosinophils, and histiocytes [39]. In addition, in Matson’s study, the heart biopsy revealed widespread lymphoplasmacytic infiltration with myocyte injury and necrosis. Granulation tissue and hemosiderin-laden macrophages were present, without notable eosinophils or neutrophils. Immunohistochemistry showed predominantly CD3-positive T-cells (CD4:CD8 ratio 1:2) and scattered CD20-positive B cells. No IgG4-positive lymphocytes were identified [29]. The last case report in which a biopsy was performed was that of Zhang L. et al., where the myocardial biopsy revealed lymphocytic myocarditis [39]. In Salem J.E.’s study, a muscle biopsy was performed and diagnosed as myositis [40].

The most commonly administered treatment for ICI-associated myocarditis was high-dose intravenous methylprednisolone, typically 1 gram per day for 3 to 5 days, followed by a gradual taper using oral prednisone or prednisolone at doses ranging from 1 mg/kg/day to lower maintenance doses. Several patients also received intravenous immunoglobulin (IVIG), particularly in severe or steroid-refractory cases. Additional immunosuppressive agents such as mycophenolate mofetil (MMF), ATG, infliximab, methotrexate, tacrolimus, and abatacept were used in more complex or relapsing cases [13,14,15,16,17,18,19,20,21,22,23,24,25,26,27,28,29,30,31,32,33,34,35,36,37,38,39,40,41,42].

Supportive therapies included cardiac medications such as beta-blockers (metoprolol, carvedilol), ACE inhibitors (lisinopril), diuretics (furosemide, IV diuretics), and, in some cases, inotropic support (e.g., norepinephrine) for hemodynamic instability. Rare but aggressive interventions included plasma exchange, hemodialysis, permanent pacemaker implantation, and multiple immunomodulatory cycles (e.g., repeat infliximab doses, IVIG courses, and extended MMF treatment) [13,14,15,16,17,18,19,20,21,22,23,24,25,26,27,28,29,30,31,32,33,34,35,36,37,38,39,40,41,42].

During hospitalization, patients experienced a wide range of cardiac complications. Conduction abnormalities such as prolonged PR interval, left bundle branch block (LBBB), and complete heart block were reported, with some requiring urgent transvenous pacing or permanent pacemaker implantation. Ventricular arrhythmias, including bigeminy, trigeminy, sustained and refractory ventricular tachycardia, and ventricular fibrillation, were common. Several patients progressed to pulseless electrical activity or cardiac arrest, requiring resuscitation and defibrillation. In some cases, telemetry revealed frequent premature ventricular contractions despite the absence of symptoms [13,14,15,16,17,18,19,20,21,22,23,24,25,26,27,28,29,30,31,32,33,34,35,36,37,38,39,40,41,42].

Cardiogenic shock developed in many patients, necessitating intensive care unit (ICU) admission, inotropic support, and mechanical circulatory assistance, including intra-aortic balloon pump (IABP) or extracorporeal membrane oxygenation (ECMO). LVEF frequently declined during the hospital stay, with some patients deteriorating rapidly from preserved function to an LVEF below 10%. A few patients responded to high-dose corticosteroids with gradual improvement in cardiac function and biomarker normalization, while others required second-line immunosuppressive therapies due to steroid-refractory disease [13,14,15,16,17,18,19,20,21,22,23,24,25,26,27,28,29,30,31,32,33,34,35,36,37,38,39,40,41,42].

Non-cardiac complications included acute renal failure, septic shock, and hepatic dysfunction. Some patients exhibited overlapping immune toxicities such as myositis, hepatitis, and myasthenia, as confirmed by serologic testing [13,14,15,16,17,18,19,20,21,22,23,24,25,26,27,28,29,30,31,32,33,34,35,36,37,38,39,40,41,42].

Outcomes varied, with some patients being discharged after clinical improvement, while others had prolonged hospitalizations or experienced fatal deterioration despite aggressive management. Specifically, among 45 patients with ICI-associated myocarditis, 25 (55.5%) were alive at follow-up, while 20 (44.5%) had passed away. Among the survivors, two patients remained alive with partial response (PR), three with stable disease (SD), and two were able to restart chemotherapy. One patient continued on long-term prednisone therapy, two had persistently elevated troponin levels, and one experienced three recurrences of irAEs.

In contrast, 20 patients died: four due to progression of the underlying disease (PD), three from ventricular tachycardia (VT) or pulseless VT, four from heart failure or cardiogenic shock with multi-organ failure, three from sepsis or septic shock, two from hemorrhagic or intracerebral stroke, one from acute myasthenic crisis, one from respiratory failure, and one from myocardial infarction. Overall, cardiovascular complications and infectious causes were the predominant contributors to mortality [13,14,15,16,17,18,19,20,21,22,23,24,25,26,27,28,29,30,31,32,33,34,35,36,37,38,39,40,41,42].

Specifically, mortality differed numerically across ICI classes, with deaths occurring in 14 of 33 PD-1 inhibitor cases (42%), 2 of 9 patients receiving combined PD-1 plus CTLA-4 blockade (22%), and 1 of 4 patients treated with PD-L1 inhibitors (25%). Overlap syndromes involving myositis, myopathy, or myasthenia gravis were identified in 11 of 46 cases (24%), and mortality was similar between overlap and non-overlap presentations (36% vs. 37%, respectively). Second-line immunosuppressive therapy—defined as the use of agents such as intravenous immunoglobulin, plasmapheresis, rituximab, or other steroid-sparing immunomodulators—was administered in 17 cases (37%). Patients who received second-line therapy had a numerically lower mortality (4/17, 24%) compared with those managed with corticosteroids alone (13/29, 45%). Additionally, fatal cases tended to occur in older individuals (median 73 vs. 66.5 years) and presented earlier following ICIs initiation (median 21 vs. 29 days). These patterns should be interpreted as descriptive and hypothesis-generating only, given the small numbers, heterogeneous reporting, and inherent publication bias of case-based data.

Table 3 summarizes the most common and less common clinical features, ECG findings, and imaging results observed in patients with immune checkpoint inhibitor–associated myocarditis.

## 5. Discussion

Myocarditis is a very rare but severe irAE associated with ICI therapy. In this report, we present a case from our hospital and conducted a literature review to better understand this uncommon yet serious side effect and its management. Our review of 44 cases revealed that most patients were older males with a mean age of 68 years, often presenting with comorbidities such as hypertension and diabetes, but notably without prior autoimmune diseases. Lung cancer was the most common underlying malignancy, and pembrolizumab was the most frequently implicated ICIs. The onset of ICI-associated myocarditis varied widely, ranging from 3 days to more than 2 years after treatment initiation, with an average onset of 67 days when excluding late cases. Clinical presentations included common cardiac symptoms such as dyspnea and chest pain, as well as neurological manifestations indicating overlap syndromes. ECG findings were diverse, spanning from mild abnormalities to severe arrhythmias. Cardiac imaging demonstrated variable myocardial damage, and elevated troponin levels were frequently observed. Importantly, distinguishing ICI-associated myocarditis from acute coronary syndrome remains challenging because patients often present with chest pain, ST-segment abnormalities, and markedly elevated troponin; however, the absence of coronary stenosis and the presence of conduction disturbances or myositis should raise suspicion for ICIs involvement. For this reason, early coronary angiography and cardiac MRI are essential to ensure that appropriate treatment can begin on time [12,13,14,15,16,17,18,19,20,21,22,23,24,25,26,27,28,29,30,31,32,33,34,35,36,37,38,39,40,41].

Several organizations, including the National Comprehensive Cancer Network (NCCN), the ASCO, the ESMO, and the International Cardio-Oncology Society (IC-OS), have recently published guidelines aimed at standardizing the diagnosis and management of ICI-associated toxicities. A number of factors have been linked to a higher risk of severe myocarditis or cardiac events during ICI therapy, including age, smoking, and pre-existing conditions such as diabetes or hypertension. Troponin levels are also important, as higher values correlate with worse cardiac outcomes. Although troponin is widely used to detect cardiac injury and guide clinical decisions, it is not specific: levels may rise due to cancer progression, anemia, sepsis, or other immune-related toxicities. As a result, while troponin can be helpful when interpreted alongside other findings, it has limited sensitivity and specificity, and false positives are common. Some studies have suggested threshold values for high-sensitivity troponin that may improve predictive accuracy, but larger cohorts are still needed. Taken together, these documents agree on the principles of rapid recognition, ICIs discontinuation, and prompt high-dose corticosteroids, but differ in how aggressively they recommend baseline screening, serial troponin/ECG monitoring, the routine use of cardiac MRI and biopsy, and the choice and timing of targeted second-line therapies such as abatacept or JAK inhibitors—underscoring the need for harmonized, data-driven algorithms [9,10,11]

Lung cancer is more often linked to ICI-associated pericarditis, while myocarditis and vasculitis are seen more often in melanoma. Female sex, autoimmune diseases, and pre-existing heart problems such as acute coronary syndrome or heart failure also increase the risk. The 2022 ESC guidelines recommend that high-risk patients receive baseline and ongoing monitoring with ECG, echocardiography, cardiac biomarkers, and natriuretic peptides during treatment. Early detection, rapid steroid treatment, and careful monitoring are essential [10].

In our review, predictors of worse outcomes included early-onset myocarditis, severe arrhythmias, quickly rising troponin levels, biventricular dysfunction, and overlap with myositis or myasthenia. Distinguishing ICI-related myocarditis from acute coronary syndrome is often difficult because symptoms can be similar, with chest pain, ST-changes, and high troponin. However, the absence of coronary artery blockage and the presence of conduction problems or myositis should increase suspicion for ICI-related myocarditis [12,13,14,15,16,17,18,19,20,21,22,23,24,25,26,27,28,29,30,31,32,33,34,35,36,37,38,39,40,41].

Immediate cessation of ICI therapy and early use of high-dose intravenous steroids are recommended. However, the timing, escalation, and choice of second-line therapy remain inconsistent across guidelines. In our case, the stepwise transition from methylprednisolone to mycophenolate and then to abatacept was prompted by clinical deterioration despite steroid treatment, in line with current recommendations for steroid-refractory myocarditis. The rapid progression in this case underscores the importance of close monitoring and early recognition of patients who may not respond to steroids alone [9,10,11].

The exact mechanisms underlying ICI-associated myocarditis remain unclear, but several pathways have been proposed. A key hypothesis involves the upregulation of PD-L1 in cardiomyocytes, which may act as a novel antigen triggering T-cell responses. After ICI therapy, especially with anti-PD-1/PD-L1 or anti-CTLA-4 combinations, studies have shown infiltration of CD4^+^ and CD8^+^ T-cells into the myocardium, along with high PD-L1 expression in damaged cardiac tissue. This suggests a disruption of immune tolerance leading to immune-mediated cardiac injury. Moreover, shared antigens between tumors and cardiac tissue, such as desmin and troponin, may cause cross-reactivity, where T cells activated against tumors inadvertently attack the heart [42,43,44,45].

One proposed mechanism is the presence of pre-existing T cells reactive to cardiac antigens such as MyHCα. These cells may escape deletion because the antigen is poorly represented in thymic epithelial cells, leaving a pool of autoreactive T cells that can become activated once ICIs lift inhibitory signals. ICIs also broaden the TCR repertoire, which has been associated with severe immune-related toxicities, suggesting both the activation of latent autoreactive clones and the expansion of new ones [42,44,45].

Furthermore, systemic immune activation from ICI therapy can increase circulating inflammatory cytokines (e.g., IFN-γ, TNF-α, IL-1), which may exacerbate off-target inflammation. Autopsy and biopsy studies consistently show clonal expansion of T cells and infiltration of macrophages (CD68^+^) in the myocardium, supporting a T cell-driven process. CD4^+^ T-helper subsets (Th1, Th17) contribute by secreting pro-inflammatory cytokines, while CD8^+^ T cells exert cytotoxic effects, directly damaging cardiomyocytes. Altogether, these mechanisms reflect a complex immune response in which ICI-induced activation of the immune system leads to unintended and often severe cardiac inflammation [45,46,47,48,49,50,51].

These mechanisms are not mutually exclusive. Loss of tolerance, antigen mimicry, and cytokine-mediated inflammation likely act together, amplifying cardiac immune infiltration, primarily by T cells and macrophages, and driving tissue injury. This interaction between the tumor, the immune-boosting effect of ICIs, and cardiac susceptibility helps explain why myocarditis incidence is markedly higher in ICI-treated patients than in the general population [45,46,47,48,49,50,51].

Histological examination of human myocardial biopsies consistently demonstrates infiltration of CD4^+^ and CD8^+^ T cells and macrophages within cardiac tissue, often with elevated PD-L1 expression on injured cardiomyocytes. These findings align with animal studies showing PD-L1 upregulation as a regulatory mechanism to limit immune-mediated damage. The presence of clonal T cells targeting shared antigens across the heart, skeletal muscle, and tumor tissue, along with elevated inflammatory mediators such as IFN-γ and TNF-α, further supports the immune-driven pathogenesis of myocarditis related to ICI therapy [19,22,23,24,28,29,30,33,38,39].

Clinically, ICI-associated cardiotoxicity is classified into four grades based on symptoms, biomarkers, and imaging findings. Management requires inpatient care with telemetry monitoring, cardiology consultation, and immediate discontinuation of ICIs [10]. Treatment mainly consists of high-dose corticosteroids, with additional immunosuppressants and supportive care for severe cases. Specifically, the ASCO guidelines suggest using abatacept (a costimulatory molecule blocker) or alemtuzumab (a CD52 blocker) as additional immunosuppression in life-threatening cases [11]. Despite treatment, mortality remained high, emphasizing the need for early diagnosis and multidisciplinary management of this serious complication [12,13,14,15,16,17,18,19,20,21,22,23,24,25,26,27,28,29,30,31,32,33,34,35,36,37,38,39,41].

It seems that broad-spectrum immunosuppressants target multiple immune cells and carry a high risk of side effects, with unclear effects on the CTLA-4 and PD-1/PD-L1 pathways. In contrast, molecules like abatacept (CTLA4-Ig) block CD28–B7–mediated T-cell co-stimulation at the dendritic cell level, acting upstream of CTLA-4 and PD-1/PD-L1. This targeted approach may induce rapid T-cell inactivation with fewer off-target effects and potentially reverse immune checkpoint inhibitor–induced responses [40,52].

Specifically, the study by Salem J.E. demonstrated that prompt administration of high-dose abatacept, guided by real-time CD86 receptor occupancy monitoring and combined with ruxolitinib, corticosteroids, and active respiratory muscle management, significantly reduced ICI-associated myotoxicity–related mortality in patients with ICI-associated myocarditis. With this approach, mortality decreased to approximately 3%, compared with the 20–60% reported in historical cohorts worldwide and the 60% observed in the first 10 cases of their own cohort who did not receive this targeted treatment and screening [53].

## 6. Future Directions

ICI-associated myocarditis remains a rare but highly lethal complication, and its unpredictable onset, heterogeneous presentation, and rapid clinical deterioration continue to challenge clinicians. Our case and literature review highlight the persistent gaps in early recognition, risk stratification, and optimal therapeutic sequencing. Current guidelines emphasize early suspicion, immediate ICI discontinuation, and prompt high-dose corticosteroids, yet real-world outcomes show that many patients remain refractory to standard therapy and that mortality remains substantial. Emerging data suggest that targeted immunomodulators such as abatacept may provide faster and more effective T-cell inactivation than broad immunosuppression alone, but prospective evidence is urgently needed [40,53]. Future directions should prioritize standardized diagnostic algorithms, validated risk-stratification tools, and cardio-oncology pathways that enable rapid multidisciplinary decision-making. Prospective registries, harmonized diagnostic criteria, and integrated cardio-oncology pathways are essential to improve early detection and management. A multidisciplinary approach, linking oncology, cardiology, rheumatology, and critical care, will be central to advancing patient outcomes and ensuring that the growing use of ICIs does not come at the cost of preventable cardiotoxicity [10].

## Figures and Tables

**Table 1:** Timeline of the patient’s clinical course, summarizing key events, biomarker trends, ECG changes, therapeutic interventions, and major complications from presentation to outcome.

Day	Key Events	Biomarkers	ECG Findings	Treatment	Complications
0	Presentation with palpitations and fatigue; admitted.	Troponin 13,983 pg/mL; CK 7166 IU/L	Sinus tachycardia, new RBBB	Diagnostic workup	—
1	Cardiac MRI confirms myocarditis.	Elevated	RBBB persists	Methylprednisolone 1 g/day	—
3	No troponin improvement (steroid-refractory).	Persistently high	Sinus tachycardia	MMF initiated	—
6	Clinical deterioration; LVEF 35%.	Rising	Ventricular arrhythmias, blocks	Amiodarone, lidocaine	VT, biventricular dysfunction
7–8	Hemodynamic collapse.	Elevated	VT persists	Intubation, vasopressors, IABP	Cardiogenic shock
8	Severe refractory myocarditis.	Very high	—	Abatacept IV	—
9–14	Progressive organ dysfunction.	—	—	Renal replacement therapy	AKI, sepsis
15	—	—	—	—	**Death from septic shock**

**Table 2:** Characteristics of included case reports. CK: Creatine Phosphokinase, CK-MB: Creatine Kinase-MB.

Author	Gender/Age	Μedical History	Cancer	ICIs	Onset (Days)	Symptoms	Electrocardiography	Troponin μμg/L/CK U/L, CK-MB ng/mL/BNP (pg/mL)	Transthoracic Echocardiography at EMU	Cardiac MRI	Management	Hospitalized	Outcome
Arangalage D. [12]	F/35	Neg	Melanoma	Ipilimumab/Nivolumab	15	NA	RBBB with ST elevation.	210/11.25/NA	LVEF 50%	Positive	IV methylprednisolone 1 g/day and IV immunoglobulin, followed by 3 days of plasma exchange, then tacrolimus.	Refractory ventricular tachycardia required emergency circulatory support; LVEF improved from 10% to 35%.	Alive
Chen Y. [13]	M/77	Neg	Chondroma	Sintilimab	21	Chest tightness, dyspnea, and upper eyelid ptosis.	ST depression in I, II, aVF, V4, V5; T wave changes in I and aVL.	1.5/706/140/581.8	LVEF 61% with enlarged left atrium and impaired LV diastolic function.	Positive	Methylprednisolone started at 480 mg/day for 5 days, tapered to 40 mg/day over 4 weeks.	CK and cTnT levels decreased during taper; abdominal infection occurred 15 days after treatment initiation.	Passed away
Cohen M. [14]	F/69	Hypertension	Lung	Camrelizumab	20	Palpitations	Sinus tachycardia, atrial premature beats, atrial tachycardia, and ST depression in leads I, II, and aVF.	0.952/NA/81.8/321	LVEF 59% with enlarged left atrium, impaired LV diastolic function, and normal systolic function.	NA	Methylprednisolone 240 mg/day for 5 days, reduced to 120 mg/day over 2 weeks, then switched to oral prednisolone 40 mg/day.	Clinical improvement noted with a decline in myocardial biomarkers.	Passed away due to respiratory failure
Compagnone M. [15]	M/77	Hypertension, DVT, cellulitis, and right eye melanoma. resection	Lung	Pembrolizumab	63	None	New onset RBBB, premature ventricular contractions, ST elevation in V3–V5, and new intraventricular block.	37.81/NA/NA/NA	LEVF 55–60%	NA	Methylprednisolone 1 g, carvedilol, atorvastatin, amiodarone, and ATG.	Developed stable sustained ventricular tachycardia with hypotension; LVEF 20–25%.	Passed away due to pulseless VT
Frigeri M [16]	F/70	Neg	Ovarian	Pembrolizumab	150	Chest pain, syncope, dyspnea, and recent fever.	ST elevation in V1 and V2 leads.	1760/NA/NA/NA	Severe LV systolic dysfunction with rounded apical adherent lesions.	Positive	Inotropic support with norepinephrine, high-dose methylprednisolone, and low-molecular-weight heparin for intraventricular thrombus.	Worsening clinical condition.	Passed away due to refractory cardiogenic shock with multiple organ failure
Fukumitsu M. [17]	F/76	Neg	Lung	Nivolumab	420	Bibasilar rales and lower limb edema.	NA	32.447/NA/NA/2674	LVEF 15% with multiple apical thrombi.	Positive	Methylprednisolone 5 mg/kg/day started on day 4, followed by plasmapheresis and IVIG 1 g/kg. On day 6, infliximab 5 mg/kg and inotropic support were given.	Cardiogenic shock developed, and she was transferred to the intensive care unit, where her LVEF dropped to less than 10%. ECMO was initiated, and an IABP was inserted.	Passed away
	M/48	NA	Lung	Nivolumab/Ipilimumab	7	Fever, elevated CRP, and hypotension.	No significant changes	Normal/Normal/NA/NA	LVEF 58%	Positive	Vasoconstrictors, methylprednisolone 1 g/day for 3 days, MMF, and IVIG.	Worsening clinical condition.	Passed away
Ansari-Gilani K. [18]	M/83	Hypothyroidism, hypertension	Renal	Nivolumab	28	Abdominal pain, right facial droop, and generalized weakness.	PVCs and ST elevation	68.49/NA/NA/NA	LVEF 35%, no regional wall motion abnormalities, small pericardial effusion.	Positive	Steroids	Hospitalized for 3 days.	Passed away
	F/78	Hyperlipidemia, depression/anxiety	Melanoma	Nivolumab/Ipilimumab	18	Blurred vision, malaise, and difficulty swallowing.	T-wave inversion	47.62/NA/NA/NA	T-wave inversion/	Negative	Steroids and plasmapheresis.	Pulseless electrical activity, resuscitated, then transitioned to comfort care.	PD
	M/81	Hypertension, COPD, AF	Melanoma	Nivolumab/Ipilimumab	25	Watery diarrhea, chest pain, and frequent PVCs.	Frequent PVCs and bradycardia.	1.44	LVEF 45%, no regional wall motion abnormalities.	Positive	High-dose steroids	Clinical improvement led to hospital discharge.	PD
Hu Y. [19]	M/60	Diabetes, hypertension, CAD	Lung	Sintilimab	22	None	Normal sinus rhythm with no ST changes.	0.303/106/7/217	LVEF 67.6%, LV diastolic dysfunction (grade II), GLS −15.8%.	Negative	IV methylprednisolone (~1 mg/kg/day) for 3 days, then tapered.	Clinical improvement led to hospital discharge.	Alive with elevated troponin
Huertas R.M. [20]	M/80	Hypertension	Renal	Nivolumab	>60	Severe asthenia and poor pain control due to subcutaneous tumor infiltration.	New-onset atrial fibrillation and LBBB.	19/1853/NA/1413	No change from 4 months prior: preserved LV systolic function with mild concentric hypertrophy and desynchrony.	NA	IV methylprednisolone 2 mg/kg/day.	Intensive therapy in Oncology and Cardiology units.	Passed away due to heart attack
Kurnik M. [21]	M/77	Hypertension, diabetes, neuropathic pain in lower extremities.	Mesothelioma	Ipilimumab/Nivolumab	22	Worsening dyspnea for 3 days, palpitations, increasing weakness, and appetite loss.	Arrhythmic tachycardia with wide QRS and Q waves in inferior and anterior leads.	3256/61.65/2.21/NA	Normal ventricular diameters, normal RV function, severely reduced LV function.	NA	Methylprednisolone 250 mg bolus, followed by 1000 mg IV bolus, IVIg, and hemodialysis.	Transferred to ICU with septic shock, hemodynamic instability, impaired cardiac function, elevated myoglobin and troponin, acute anuric renal failure, worsening septic shock, and sharp rise in liver transaminases.	Passed away due septic and cardiogenic shock.
Le Bras P. [22]	M/76	Hypertension, dyslipidemia, HCV, hepatic fibrosis.	Hepatocytic	Atezolizumab	147	Intermittent chest tightness, dyspnea, and right calf pain for 7 days.	Repolarization abnormalities with 2–3 mm right precordial elevation, left precordial depression, LV hypertrophy, and LBBB.	37/Normal/NA/2952	Diffuse hypokinesia, worse in inferior and lateral walls, LVEF 35%, with concentric LV hypertrophy.	Positive	IV diuretics followed by oral therapy and cardioprotective treatments.	Clinical improvement led to hospital discharge.	Alive with elevated troponin- Rechallenge of ICI
Izumi R. [23]	M/72	Hypertension, diabetes	Renal	Avelumab/Nivolumab	18	Presyncope	Sustained VT with wide QRS and ST elevation in V1–V3.	1871/5724/96/456.2	LVEF 40%	NA	Methylprednisolone 1 g/day for 3 days, then prednisolone 1 mg/kg daily.	Associated irAEs: hepatitis and myositis.	Passed away due to VT
	M/69	Hypertension	Prostate	Pembrolizumab	839	Fever, vomiting and chest pain.	CAVB with wide QRS, ST depression in I, aVL, V1–V6, and ST elevation in aVR.	48.118/2450/365/921.7	Diffuse LV hypokinesis with LVEF of 17%.	NA	Methylprednisolone 1 g/day for 3 days, then prednisolone 10 mg daily.	In cardiogenic shock, IABP and temporary transvenous pacing were initiated.	Heart failure and multiple organ failure
	M/63	None	Lung	Atezolizumab	13	Fever and chest pain.	Wide QRS with ST elevation in V1–V4.	15.561/3859/61/388.9	Severe diffuse LV hypokinesis, LVEF 10%.	NA	Methylprednisolone 1 g iv daily for 3 days.	Worsening clinical condition.	Passed away due to intracerebral
	F/76	Hypertension, IHD	Lung	Atezolizumab	11	Fatigue and dyspnea.	Poor R-wave progression in V1–V4 and flat T-waves with normal QRS duration.	0.358/101/5/1357.3	Diffuse LV hypokinesis, LVEF 10%.	NA	Methylprednisolone 1 g/day for 3 days, then prednisolone 1 mg/kg daily.	Transferred to rehab on day 35; discharged home after 3 months.	Alive
Lewis I. R. [24]	/F57	NA	Lung	Pembrolizumab	>2.5 yrs	Cardiac arrest.	VT	0.098/156/NA/NA	Heart failure with a reduced LVEF = 36%.	Positive	IV prednisone 1 mg/kg daily.	The patient received an implantable cardioverter-defibrillator for secondary prevention, and pembrolizumab was permanently stopped.	Alive
Liang S. [25]	M/77	NA	Chondroma	Sintilimab	21	Acute onset of dyspnea.	Sinus rhythm with ST depression (0.5–1.0 mm) in I, II, aVF, V4–V6; T wave inversion in aVL; Q wave in III.	1.29/NA/140.7/581.8	Normal LV systolic function, LVEF 61%.	Positive	IV methylprednisolone taper over 31 days (160 mg → 80 mg → 40 mg), then oral prednisone 50 mg daily.	Antibodies against AChR and Titin were positive. The patient’s vital signs stabilized gradually and he was discharged.	Alive
Liu X. [26]	F/45	Neg	Gastric	Sintilimab	28	Chest pain and palpitations.	ST elevation in multiple leads.	5574/NA/96.7/111.9	Dilated RV (4.5 cm) and RA (4.6 cm) with tricuspid regurgitation; LVEF preserved at 68%	NA	IV methylprednisolone 120 mg and IVIG.	Patient had sudden seizures, respiratory arrest, and VF; CPR, epinephrine, and defibrillation restored rhythm.	Alive
Liu Z. [27]	F/60	Diabetes	Pancreatic	Camrelizumab	42	Progressive muscle weakness and dyspnea for 4 days.	Short VT burst, accelerated idioventricular rhythm, and ST-T abnormalities.	2.2/NA/NA/3596	New wall motion abnormality, LVEF 34%. Ask ChatGPT-4.1	Positive	IV methylprednisolone 1 g/day for 3 days, IVIG 10 mg/day, MMF started at 500 mg/day increased to 1 g/day, with methylprednisolone 120 mg/day added from days 5 to 9.	Patient had recurrent Adams–Stokes episodes with VF/VT on ECG, regaining consciousness after repeated CPR and defibrillation.	Alive
Mariniello M. [28]	F/40	Hashimoto’s thyroiditis	Lung	Nivolumab and Ipilimumab	30	Cough, dyspnea, and right chest pain.	Sinus rhythm with normal AV conduction, ST elevation in inferolateral and V3–V6 leads, and RBBB.	13.963/6388	Normal LVEF, hyperechoic inferolateral wall, mild anterior pericardial thickening and detachment.	Positive	IV methylprednisolone 1 g/day for 5 days, MTX 7.5 mg/week, and pyridostigmine.	Diagnosis: immune-related myocarditis–myasthenia–myositis syndrome.	Alive and chemotherapy re started.
Matson R.D. [29]	M/55	COPD	Lung	Nivolumab	43	Lethargy and dyspnea.	Wide-complex VT with cool limbs.	14.43/NA/NA/NA	Dilated right ventricle and atrium with hepatic vein reflux, indicating right heart failure.	NA	Hemodialysis.	Diagnosed with acute decompensated right heart failure causing cardiogenic shock and multi-organ failure, including likely perfusion-related acute kidney injury.	Passed away
Miyauchi Y. [30]	M/71	Hypertension, diabetes, hyperuricemia	Renal	Nivolumab and Ipilimumab	25	Asymptomatic with mild sustained CK elevation.	ST elevation in all leads.	10068/NA0/23480/15964	Akinetic apex, hypokinetic septum, LVEF 50%.	Positive	IV methylprednisolone 500 mg for 3 days, then prednisolone 1 mg/kg.	Patient developed cardiogenic shock with widespread cardiac akinesis sparing posterior wall, admitted to ICU for support, and had ventricular fibrillation.	Alive with long-term prednisone therapy (15 mg/day)
Monge C. [31]	M/79	AF	Colon	Nivolumab	56	Blurred vision with upper back pain and stiffness.	AF with QTc prolongation (514 ms) and stable left anterior fascicular block.	0.209/3200/65.7/3066	LVEF 65%, enlarged left atrium, dilated right ventricle, and pulmonary artery pressure 45 mmHg.	Positive	Methylprednisolone 1 mg/kg daily.	Patient discharged on multi-week oral prednisone taper after cardiac enzymes normalized on day 4; PROSTVAC continued for 3 months, nivolumab stopped.	Alive
Man X. [32]	M/65	Hypertension, diabetes, CAD.	Urothelial	Toripalimab	31	Chest discomfort, weakness, and palpitations.	Sinus tachycardia with ST elevation.	0.750/7420/74.20/Normal	NA	NA	Methylprednisolone	NA	Alive
	F/67	Hypertension	Lung	Sintilimab	21	Dyspnea, chest discomfort, myalgia, weakness, and ptosis.	ST elevation with RBBB.	10.500/11601/194/3050	NA	NA	Methylprednisolone	NA	Passed away due to PD
	F/68	Hypertension, Diabetes.	Urothelial	Toripalimab	25	Ptosis, weakness, and myalgia.	Sinus tachycardia with ST elevation.	104/5346/0.949/Normal	NA	NA	Methylprednisolone, IFX, MMF, IVIG	NA	Alive
	M/68	Hypertension, Diabetes, CAD	Renal	Toripalimab	22	Dyspnea, myalgia, and weakness.	ST elevation	64.30/NA/15/2700	NA	NA	Methylprednisolone	NA	Alive
	F/65	Neg	Lung	Pembrolizumab	600	Palpitations and dyspnea.	Sinus tachycardia with ST elevation.	NA/NA/6.37/6590	NA	NA	Methylprednisolone and IVIG	NA	Alive
Naganuma K. [33]	M/74	Resected bladder cancer	Gastric	Nivolumab	3 months post-nivolumab discontinuation for irAE (day 240).	Fatigue and nausea.	ST elevation in II, III, aVF with subsequent complete AV block.	17971/402/NA/NA	LVEF 73%, no pericardial effusion.	NA	Started prednisolone 60 mg (1 mg/kg), then methylprednisolone 1 g/day for 3 days, IVIG 1 g/kg for 2 days, and plasma exchange.	By day 3 post-admission, EF dropped from 73% to 10%, leading to a diagnosis of fulminant myocarditis.	Passed away due to sepsis
Nair D.P. [34]	F/64	COPD, hypertension, diabetes, hyperlipidemia	Colon	Pembrolizumab	210	Dyspnea.	Non-specific ST elevations in anteroseptal leads without reciprocal changes.	0.35/687/NA/25	LVEF 25%, hyperdynamic basal segments, severe hypokinesis elsewhere.	Positive	IV methylprednisolone 1 g/day, then prolonged oral prednisone taper.	-	Alive
Yang Y. [35]	F/51	Neg	Breast	Pembrolizumab	3	High fever, mild dyspnea, and systemic rash.	NA	NA/NA/NA1991	NA	NA	Methylprednisolone tapered from 80 mg/day to 60 mg/day, then gradually reduced to 8 mg/day and discontinued.	-	Alive with SD
Sakaguchi H. [36]	M/73	NA	Lung	Pembrolizumab	20	Proximal muscle pain	Sinus rhythm with RBBB	0.697/21322/NA/NA	No heart failure signs.	Positive	Treated with IV methylprednisolone 1 g/day for 6 days, IVIG 20 g/day for 5 days, and tacrolimus 2 mg/day, followed by oral prednisolone.	-	Alive with PR
	M/67	NA	Renal	Ipilimumab/Nivolumab	21	Palpitations	AF with tachycardia	0.143/NA/NA/NA	NA	NA	Prednisolone 2 mg/kg, then switched to methylprednisolone 1 g/day for 3 days, followed by 60 mg/day (1 mg/kg) and IVIG 20 g/day for 5 days.	-	PD
Tan S. [37]	M/62	Hypertension, diabetes	Lung	Pembrolizumab	35	Dizziness, bilateral ptosis, and eye movement abnormalities.	Normal	442.1/75.21/NA/Normal	LVEF 68%	NA	Methylprednisolone (80 mg/day, then 500 mg/day for 7 days), IVIG, MMF, and infliximab, followed by tapering oral prednisone (50 mg/day).	-	PD
	F/57	Neg	Lung	SHR-1701	84	One week of dyspnea.	NA	299.5/77.92/NA/Normal	Enlarged left atrium, thickened interventricular septum, widened ascending aorta and pulmonary artery, with mild LVEF decrease from 73% to 63%.	NA	Initial treatment with methylprednisolone 80 mg/day for 9 days with tapering. On second admission: methylprednisolone 500 mg/day for 3 days, MMF 1 g/day for 11 days, IVIG 20 g/day for 3 days, and infliximab on days 31, 45, and 73.	She was readmitted on day 16 with dyspnea and elevated troponin (210 ng/L), and diagnosed with steroid-refractory ICI-induced myocarditis.	Alive with PR and re-started of chemotherapy
Zasok O.I.B. [38]	F/53	NA	Renal	Ipilimumab/Nivolumab	77	Dizziness	Anterolateral ST-segment elevation	2469/NA/NA/8840	Severe biventricular systolic dysfunction with LVEF of 30%.	Positive	Methylprednisolone 1 g/day for 3 days, MMF, then prednisone 1 mg/kg/day.	The asymptomatic patient had frequent PVCs on telemetry, later developing refractory VT and cardiogenic shock.	Alive
Zhang L. [39]	M/69	Hypertension, diabetes	Renal	Atezolizumab	42	Fatigue and dyspnea.	Sinus tachycardia with non-specific ST/T changes.	1.25/NA/NA/NA	LVEF of 20%	Positive	AATG	T The patient rapidly deteriorated and developed cardiogenic shock, requiring inotropic support.	Alive
Salem J.E. [40]	F/66	NA	Lung	Nivolumab	42	Ptosis, diplopia, subacute painful weakness of proximal muscles.	Repolarization abnormalities	1616/NA/NA/4172	NA	Positive	Methylprednisolone (500 mg/d for 3d), plasmapheresis on D7->intravenous abatacept (500 mg q2w beginning on D17	Abatacept led to rapid improvement in cardiac and muscle symptoms, with stable ejection fraction. The patient was discharged after 7.5 weeks, and imaging showed no tumor progression.	Alive with stable disease
Agrawal N. [41]	M/73	NA	Mesothelioma	Pembrolizumab	32	2-day progressive dyspnea, weight gain, and fatigue.	Alternating RBBB/LBBB, asystole episode, and third-degree block with junctional escape rhythm.	8.3/6124/99,1/NA	LVEF 50–60%, mild LVH, mild to moderate atrial enlargement, mild aortic, mitral, and tricuspid regurgitation.	NA	Permanent pacemaker, lisinopril, metoprolol, furosemide, and two doses of oral prednisolone 60 mg/day.	Worsening clinical condition.	Passed away due to acute myasthenia crisis
	M/64	Hypertension, Castleman disease, and prostate cancer.	Melanoma	Pembrolizumab	28	2 weeks of fatigue, weakness, myalgia, diplopia, and reduced eye movement.	RBBB with left anterior fascicular block.	0.78/1681/159/NA	Normal LVEF, no wall motion abnormalities.	Negative	1 g IV methylprednisolone for 3 days, then oral prednisolone 150 mg/day from day 4, with 5 days of IVIG and pyridostigmine.	-	Alive with SD
	M/89	Diabetes, hypertension, dyslipidemia, and atrial fibrillation.	Melanoma	Pembrolizumab	12	Weakness, myalgia, and resting dyspnea.	RBBB	5.78/964/104.4/105	Right ventricular dilation with normal function; hyperdynamic left ventricle with preserved wall motion.	NA	1 g IV methylprednisolone daily, followed by oral prednisone 60 mg twice daily, and 3 days of ATG 125 mg/day.	Complete heart block; urgent transvenous pacing inserted.	Passed away due to septic shock
	F/65	Hypertension, mitral valve repair, laryngectomy.	Lung	Nivolumab	6	Exertional dyspnea.	NA	0.12/NA/2.2/764	Diffuse global hypokinesis with LVEF 25–30%.	Positive	1 g IV methylprednisolone daily, diuretics, oxygen, low-dose lisinopril and carvedilol, midodrine, and 2 doses of ATG.	Cardiogenic shock with ventricular bigeminy and trigeminy; EF 60-65%.	Alive with 3 times recurrence of irAEs
	M/67	Coronary artery bypass, peripheral arterial disease, hypertension, diabetes.	Melanoma	Nivolumab	42	Left chest pain with palpitations.	Sinus rhythm with lateral ST depressions.	1.55/NA/NA/NA	NA	Positive	1 g IV methylprednisolone, followed by prednisone 80 mg twice daily for 5 days with tapering; infliximab.	Mild biventricular dilation, reduced RV function, bi-atrial enlargement, LVEF 55%.	Alive

**Table 3:** Most and least common features of ICI-associated myocarditis. CAVB: Complete atrioventricular block, VT: ventricular tachycardia, LVEF: Left Ventricular Ejection Fraction.

Category	Most Common	Less Common
Presenting Symptoms	Dyspnea, fatigue, chest pain/discomfort, myalgia, palpitations Neurological symptoms (ptosis, diplopia, blurred vision, weakness)	Cardiac arrest, presyncope, asymptomatic
ECG Findings at Presentation	ST-segment elevation in various leads, sinus tachycardia, and newly diagnosed bundle branch block	CAVB, VT, wide QRS arrhythmias, QT prolongation, alternating bundle branch blocks, asystole, third-degree heart block with junctional escape rhythms
LVEF and Echocardiogram Findings	Preserved or mildly reduced LVEF (50–60%)	Severe LVEF (10–35%)
MRI Findings	Majority cases unavailable; among available, most positive for myocarditis	Negative for myocarditis

## Data Availability

The datasets generated and analyzed during the current study are available at DOI 10.5281/zenodo.17576077.
